# Elecampane as an Antiseptic

**Published:** 1885-06

**Authors:** 


### Elecampane as an Antiseptic.

Among the familiar roadside weeds of the Northern States, the rough-stemmed, yellow-flowered elecampane is as conspicuous as any. Though less aggressive and troublesome than thistles, burdocks, and some others in the list of European migrants, the elecampane is regarded with little favor by farmers, in spite of the well attested medical virtues of its roots. It is by habit a vegetable tramp—a weed ; and with the least encouragement it has traversed every highway and by-road from Maine to the Mississippi, straggling into fields and meadows wherever suitable conditions of moisture and fertility promise for it, what all tramps go for, a plenty of easily acquired food. It was originally brought to this country as a garden or door-yard plant, partly for its gaudy flowers, partly for its utility in domestic medicine ; but for many years it has been held in little esteem on either account, more thorough change of fashion, however, than for any fault of the plant. Indeed, it now appears that, like many another victim of popular neglect, the elecampane is worthy of restoration to public favor, and may in truth prove to be justly reckoned among the most useful of useful plants.

In a recent issue the *Lancet* mentions a series of articles which have appeared lately in a pharmaceutical paper of Barcelona, describing investigations which go to demonstrate that the chief active principle of the elecampane, *helenina* (from the systematic name of the plant *Inula helenium*), is one of the most powerful antiseptics known, and at the same time free from the disagreeable odor of carbolic acid, which it might well replace.

It is not clear whether *helenina* (as the *Lancet* spells it) is the *helenin* of Gerhardt ($C_{21}H_{28}O_3$), obtained by extracting the active principle of elecampane with hot alcohol, in the form of needle-shaped crystals fusing at 72 deg., or the *helanine* of later chemists ($C_{12}H_{16}O_2$), which results from repeatedly recrystallizing the crude extract and separating from it inula-camphor ($C_{10}H_{16}O$). The latter fuses at 64 deg. ; *helanine* at 110 deg. As thus purified *helanine* is described by Watts as colorless, inodorous crystals, nearly insoluble in water, and easily soluble in alcohol. In the U. S. Dispensatory this compound is described as intermediate in its properties between essential oils and camphor. Inula-camphor is isomeric with camphor, and strongly resembles menthol, or peppermint-camphor, now a fashionable remedy for headache. The essential oils nearly all possess the composition $C_{10}H_{16}$, and, as Prof. Mantegazzi showed in 1870, their oxidation when exposed to light is a powerful and convenient means of producing ozone, giving them high value as disinfectants.

Thus from what is well known of helanine and its allies, it is not surprising that it should be valuable as an antiseptic. The investigations first referred to seem to have been suggested by those of Dr. Korab, who found one part of an alcoholic solution of helanine sufficient to arrest putrefaction in ten thousand parts of urine ; also that a few drops of the solution immediately killed the organisms in ordinary infusious, and also in cultivations of tubercle bacillus

The writer in the *Boletin Farmaceutico* applied an alcoholic solution of helenina to slices of veal, which, though kept at a temperature of 28 deg. C. (82·4 deg. Fah.), remained sweet for ten days, or until completely dry. An egg beaten up with nearly a pound of water was treated with 5 grains of helenina in six times its weight of alchohol remained unchanged for six days at a temperature of 82 deg. Another egg similarly beaten up with water, without the drug, rapidly decomposed, and in twenty hours emitted a strong odor of sulphide of hydrogen. When to this solution about 8 grains of helenina were added, the offensive odor quickly disappeared, and the mixture underwent no further change.

Similar experiments with urine, meat, and beaten-up eggs were made with carbolic, boracic and salicylic acids instead of helenina; but much larger proportions of the acids were required to prevent putrefaction, and none of them was able to arrest putrefaction already begun, as the helenina had done. It was also observed that the aromatic smell of the materials from which the drug was extracted repelled all insects, even mosquitoes, from the house in which the experiments were made.

The *Lancet* adds that helenina has proved valuable in surgery as an antiseptic when carbolic acid and all other agents had failed; also that it has been given successfully in malarial fevers, and tuberculous, infantile and catarrhal diarrhœa; and that it is expected to form an excellent substitute for carbolic acid in the Listerian system of aseptic surgery. Possibly the power of the drug to kill low organisms is what has made it useful as an internal and external remedy in tetter, psora, and other diseases of the skin, as mentioned in the Dispensatory. In this country it has been chiefly used of late in chronic diseases of the lungs. It is said to be sometimes beneficial when the chest trouble is attended with weakness of the digestive organs or with general debility. The ancients employed elecampane roots very largely in medicine, and it would seem to be still more generally used in Europe than in America. If its alleged antiseptic and germicidal properties are confirmed by further tests, it is probable that the despised weed may rank with the cinchona tree in sanitary and commercial importance.

The clever definition " *Weed.*—A plant whose uses are not discovered," thus receives a new and striking illustration. Who can tell how many other old weeds are awaiting new uses, to justify their persistence in living?

Elecampane is a coarse-looking plant; the stem, rising to six feet, is furrowed, branching and downy above. The radical leaves are very large and rough, with serrated edges. The upper leaves are smaller, and embrace the stem. The flowers, which appear in July and August, are in heads, like sunflowers, and stand singly at the ends of the stem and branches. Their color is a golden yellow; odor aromatic. The stem is renewed every year; the root is perennial. The fresh root is very thick and branched, having whitish cylindrical ramifactions with thread-like fibers. The outside is brown; within, the root is whiteish, and fleshy. The agreeably aromatic odor of the root is increased by drying. The roots are dug in the fall, and are best in their second year; when older, they are apt to be woody. The dried root can be procured in almost every drug store, and might be worth trying as an agreeable and possibly efficient means of keeping apartments free from flies, mosquitoes, and other insects. The ozonizing power of the odor is likely to be valuable also in helping to destroy bad smells, even if the active principle should be less efficient than the Spanish authorities affirm in preventing putrefaction and like unsanitary processes.

It may be worth while also to encourage the growth of the plant around outhouses, ditches, and drains, instead of the now fashionable but coarser and less efflcient sunflower, for the purifying of the air and the prevention of malaria.—*Scientific American.*

